# PAMs ameliorates the imiquimod-induced psoriasis-like skin disease in mice by inhibition of translocation of NF-κB and production of inflammatory cytokines

**DOI:** 10.1371/journal.pone.0176823

**Published:** 2017-05-02

**Authors:** Rongkun Dou, Zongying Liu, Xue Yuan, Danzhou Xiangfei, Ruixue Bai, Zhenfei Bi, Piao Yang, Yalan Yang, Yinsong Dong, Wei Su, Diqiang Li, Canquan Mao

**Affiliations:** 1 School of Life Sciences and Engineering, Southwest Jiaotong University, Chengdu, China; 2 Institute of Yunnan Folk Medicine Co. Ltd, Kunming, China; 3 Institute of Forest Ecology, Environment and Protection, Chinese Academy of Forestry, Beijing, China; University of Alabama at Birmingham, UNITED STATES

## Abstract

Psoriasis is a chronic and persistent inflammatory skin disease seriously affecting the quality of human life. In this study, we reported an ancient formula of Chinese folk medicine, the natural plant antimicrobial solution (PAMs) for its anti-inflammatory effects and proposed the primary mechanisms on inhibiting the inflammatory response in TNF-α/IFN-γ-induced HaCaT cells and imiquimod-induced psoriasis-like skin disease mouse model. Two main functional components of hydroxysafflor Yellow A and allantoin in PAMs were quantified by HPLC to be 94.2±2.2 and 262.9±12.5 μg/mL respectively. PAMs could significantly reduce the gene expression and inflammatory cytokines production of Macrophage-Derived Chemokine (MDC), IL-8 and IL-6 in TNF-α/IFN-γ-induced HaCaT cells. PAMs also significantly ameliorates the psoriatic-like symptoms in a mouse model with the evaluation scores for both the single (scales, thickness, erythema) and cumulative features were in the order of blank control < Dexamethasone < PAMs < 50% ethanol < model groups. The results were further confirmed by hematoxylin-eosin staining, RT-qPCR and immunohistochemistry. The down-regulated gene expression of *IL-8*, *TNF-α*, *ICAM-1 and IL-23* in mouse tissues was consistent with the results from those of the HaCaT cells. The inhibition of psoriasis-like skin inflammation by PAMs was correlated with the inactivation of the translocation of P65 protein into cellular nucleus, indicating the inhibition of the inflammatory NF-κB signaling pathway. Taken together, these findings suggest that PAMs may be a promising drug candidate for the treatment of inflammatory skin disorders, such as psoriasis.

## Introduction

Skin is the largest body’s organ serving as the barrier between the environment and internal milieu, and involved in numerous biological processes, such as skin immune, pigmentary, epidermal and adnexal systems [1]. Skin is also a complex networks and can regulate pro- and anti-inflammatory actions, and many skin diseases are related to inflammatory response [1–3]. Among various skin problems, psoriasis is a common, relapsing and chronic skin disease affecting approximately 1–3% of the human population [4]. It is characterized by epidermal keratinocyte proliferation, parakeratosis and massive dermal infiltration of leukocytes [5]. Although genetic and environmental factors have important roles in the pathogenesis of psoriasis [6], an immune-mediated inflammatory mechanism in this disorder may also be involved in its pathogenesis by infiltration of immune cells and activation of keratinocytes [7]. Nuclear factor-κB (NF-κB) is among the key effectors in inflammatory responses related to various skin diseases, including psoriasis [8]. It initiates the inflammatory response by activating the NF­κB signaling pathways and subsequently producing inflammatory cytokines and chemokines [9]. Furthermore, the activation of NF-κB has been illustrated in lesioned psoriatic skin [10]. Thus, by inhibiting the NF­κB signaling pathway and inflammatory reactions, psoriasis may be efficiently alleviated or cured.

Currently, the commonly used drugs or methods for the treatment of psoriasis include retinoids, immunosuppressants, glucocorticosteroids, photo-therapies and bio-therapies [11]. So far, there are no satisfactory strategies for curing the disease. Traditional Chinese Medicine (TCM) may open a new avenue for the effective therapy of such diseases.

TCM have been used for thousands of years in China and Eastern countries, and are the precious treasure of Eastern civilization and healthcare. Liuwei Dihuang Pill prepared from six kinds of medical plants [12], injections extracted from Danshen (*Salvia miltiorrhiza*) [13] and berberine originally obtained from *Coptis chinensis Franch* are well-known examples and widely accepted TCM products. Although Western medicine practitioners often viewed them with skepticism, Chinese herbal medicine and plant-derived extracts/monomers have shown great potential in medical use, including the treatment for psoriasis [14,15]. Moreover, herbal medicine is regarded as relatively safe and having lower side-effects [16,17].

The natural plant antimicrobial solution (PAMs) was a complex ethanolic extract of Chinese natural and folk medicinal plants with multi-bioactive components. It is originated from an effective formula and has been used clinically in China for hundreds of years during war and peaceful time to prevent inflammation and to promote wound healing. It is cost-effective, and the delivery of PAMs by fabrification for topical use is considered to be safer and more acceptable. In 2015, PAMs was approved to be the hospital preparation by China Yunnan Food and Drug Administration (CFDA) for prevention of wound infection and festering, cell necrosis, dry gangrene and blood circulation obstacles. In addition, it was found in local application that PAMs could be curative for psoriasis and skin lesions; however, the underlying mechanism for the effects of PAMs was not clearly understood. In the present study, we quantified two functional components in PAMs and showed that PAMs could inhibit the TNF-α/IFN-γ-induced inflammatory cytokines production in HaCaT cells and ameliorate imiquimod-induced psoriasis-like skin inflammation in mice by inhibiting the translocation of p65 in NF-κB signaling pathways. These findings strongly suggested that PAMs could be a powerful candidate drug for the treatment of inflammatory disorders such as psoriasis.

## Material and methods

### Preparation of PAMs

PAMs was developed by the Institute of Yunnan Folk Medicine Co., Ltd. and produced by Yunnan Puer Danzhou Pharmaceutical Co., Ltd. (Yunnan Province, P.R. China). Briefly, dry samples of the medicinal plants including *Carthamus tinctorius*, *Lithospermum erythrorhizon*, *Solanum indicum*, and *Cymbopogon distans* (20.0, 30.0, 10.0 and 20.0 g, respectively) were milled, mixed and extracted with 90% ethanol at room temperature for 720 h, then the extracts were collected and the residues were again extracted with 90% ethanol for 360 h. The total ethanol extracts were mixed and the solution was diluted with double distilled water to 1 L (the final ethanol content was adjusted to 50%) and stored at 4 ℃. PAMs was an invent patent authorized by the State Intellectual Property Office of P. R. China in 2013(No. 201110393033.3), in addition, it was approved to be disinfectant product in 2011(China Yunnan Hygienic Disinfection Certificate [2011]No. 0004) and Hospital Preparation in 2015[China Yunnan FDA (No. Z20150009)]. The raw medicinal plants were identified by Mr. Zhiwen Wu, Manager of the Quality Control Department of the Company.

### HPLC analysis of PAMs

Standard stock solutions of hydroxysafflor Yellow A (Chengdu Must Bio-Technology CO., Ltd, Chengdu, Sichuan, China) were dissolved in 25% methanol at a concentration of 100.0 μg/mL, allantoin (Chengdu Must Bio-Technology CO.) was dissolved in 100% methanol at a concentration of 200.0 μg/mL. All of them were stored at 4℃. The standard working solutions were prepared by serial dilution of the stock solutions with 25% methanol or 100% methanol.

Chromatographic analysis was performed for simultaneous determination of the PAMs ethanol extracts using an Agilent HPLC 1260 system (Agilent, Palo Alto, CA, USA). All analytes were separated on a Waters Symmetry Shield RP18 (250mm ×4.6mm, 5μm) and maintained at 30℃. As shown in Table 1, the isocratic flow was used for the analysis of each component.

**Table 1:** HPLC parameters for the determination of the two main components in PAMs.

Component	Mobile phase	PDA wavelengths (nm)	Flow rate (ml/min)
Hydroxysafflor Yellow A	Methanol−0.4% (v/v) phosphoric acid−acetonitrile (28:70:2)	403	1.0
Allantoin	Methanol−water (40:60)	224	1.0

### Cell culture and viability assay

Human keratinocyte HaCaT cells were kindly provided by the Key Laboratory of Transplant Engineering and Immunology, Ministry of Health; West China Hospital, Sichuan University, China. The cells were cultured in Dulbecco’s modified Eagle’s medium (DMEM, HyClone, Logan, UT, USA) supplemented with 10% heat-inactivated fetal bovine serum (FBS, HyClone, Logan, UT, USA), penicillin (100.0 U/ml) and streptomycin (100.0 μg/ml) at 37℃ in a 5% CO_2_ incubator. Cell viability was measured by a Cell Counting Kit-8 method (CCK-8, KeyGEN BioTECH, Nanjing, China) according to the manufacturer’s instructions. Briefly, HaCaT cells (5.0 ×10^3^ cells/well) were incubated in a 96-well plate for 24 h and then treated with increasing concentrations of PAMs (1.0, 2.0, 3.0 and 4.0%, v/v, *n =* 6). After incubation for another 24 h, CCK-8 reagent was added to each well and the cells were incubated for an additional 2 h. The absorbance was measured at 450 nm by using a microplate reader Synergy H1/H1MF (BioTek, Winooski, Vermont, USA). The percentage of viable cells was calculated by using the following equation: cell viability (%) = (mean absorbance in test wells/mean absorbance in control wells) × 100.

### ELISA detection of cytokines

For cellular supernatant detection, HaCaT cells (1.0 ×10^6^ cells/well) were cultured in a 6-well plate. After reaching the confluent state, the cells were treated with or without 3.0% (v/v) PAMs in 1.0 mL of serum-free medium alone or along with TNF-α and IFN-γ (each 10.0 ng/mL; Peprotech, Rocky Hill, USA) for 24 h. The production of cytokines (IL-8, IL-6, IL-1β and MDC) in the supernatant was quantified with an ELISA kit (KYM, Beijing, China).

For mouse blood samples, they were collected and subjected to centrifugation at 3000 rpm (HERMLE, Germany) for 15 min and stored at -20℃ until use. Serum cytokine (TNF-α) levels were measured by ELISA kit (KYM, Beijing, China).

### RNA isolation and quantitative real time PCR (RT-qPCR) in HaCaT cells

Total RNA were isolated from the cell cultures using TRIzol reagent (Ambion, USA). Two microgram of total RNA was converted to cDNA with an iScript cDNA Synthesis kit (Aidlab Biotechnologies Co., Ltd, Beijing, China). Quantitative real-time PCR was performed using SYBR Green Master Mix (Roche, Switzerland) according to the manufacturer's instructions. The sequences of the real-time PCR primers were showed in Table 2. Melting curve analysis was used to evaluate the primer specificity of the amplification. The gene *β-actin* was used as the internal control. The relative mRNA expression of *IL-8*, *IL-6*, *MDC*, *ICAM-1* were calculated by normalizing to *β-actin*.

**Table 2:** Primer design of chemokines/cytokines in HaCaT cells.

Genes	Primer sequence	Tm
*ICAM-1*	F: 5’-CACCCTAGAGCCAAGGTGAC-3’	60°C
	R: 5’-CATTGGAGTCTGCTGGGAAT-3’	
*IL-6*	F: 5’-AGAGTAGTGAGGAACAAGCC-3’	60°C
	R: 5’-TACATTTGCCGAAGAGCCCT-3’	
*IL-8*	F: 5’-AGAGTGGACCACACTGCGC-3’	60°C
	R: 5’-GTGTTGAAGTAGATTTGCT-3’	
*MDC*	F: 5’-AGGACAGAGCATGGaTCGCCTACAGA-3’	60°C
	R: 5’-TAATGGCAGGGAGGTAGGGCTCCTGA-3’	
*β-actin*	F: 5’-CGGGAAATCGTGCGTGAC-3’	60°C
	R: 5’-CAGGAAGGAAGGCTGGAAG-3’	

### Immunofluorescence staining of HaCaT cells

HaCaT cells (5.0 ×10^3^ cells/well) were cultured in a 96-well plate. The cells were pretreated with or without 3.0% (v/v) PAMs or 1.5% (v/v) ethanol for 6 h and incubated with TNF-α and IFN-γ (each 10.0 ng/mL) for 30 min. After fixation, permeability, Triton X-100 treatment and blocked with 5.0% BSA for 1 h at room temperature, the cells were incubated overnight with the p65 antibody (dilution 1:200, AN365, Beyotime Institute of Biotechnology, Nanjing, China) at 4℃. The samples were washed with PBS twice and incubated with Cy3-conjugated secondary antibody (dilution 1:1000, P0183, Beyotime Institute of Biotechnology) for 1.5 h at 37℃. Finally, the cells were stained with 100.0 μl DAPI for 4 min, washed twice with PBS and observed using a fluorescence microscope (Olympus, Tokyo, Japan). The translocation rate was counted from all fields.

### Mouse and ethics statement

Female BALB/c mice were purchased from Chengdu Dashuo biological Co., Ltd (Chengdu, China). The mice were at 6–8 weeks of age between 20.0 to 22.0 g under specific pathogen-free conditions and supplied with food and water *ad libitum*. The experiments were performed according to the Animal Care and Use Committee guidelines. The protocol was approved by the Committee on the Ethics of Animal Experiments of Southwest Jiaotong University. All mice were sacrificed by cervical dislocation and all efforts were made to minimize suffering.

### Imiquimod-induced skin-inflammation and evaluation

**Fig 1:**
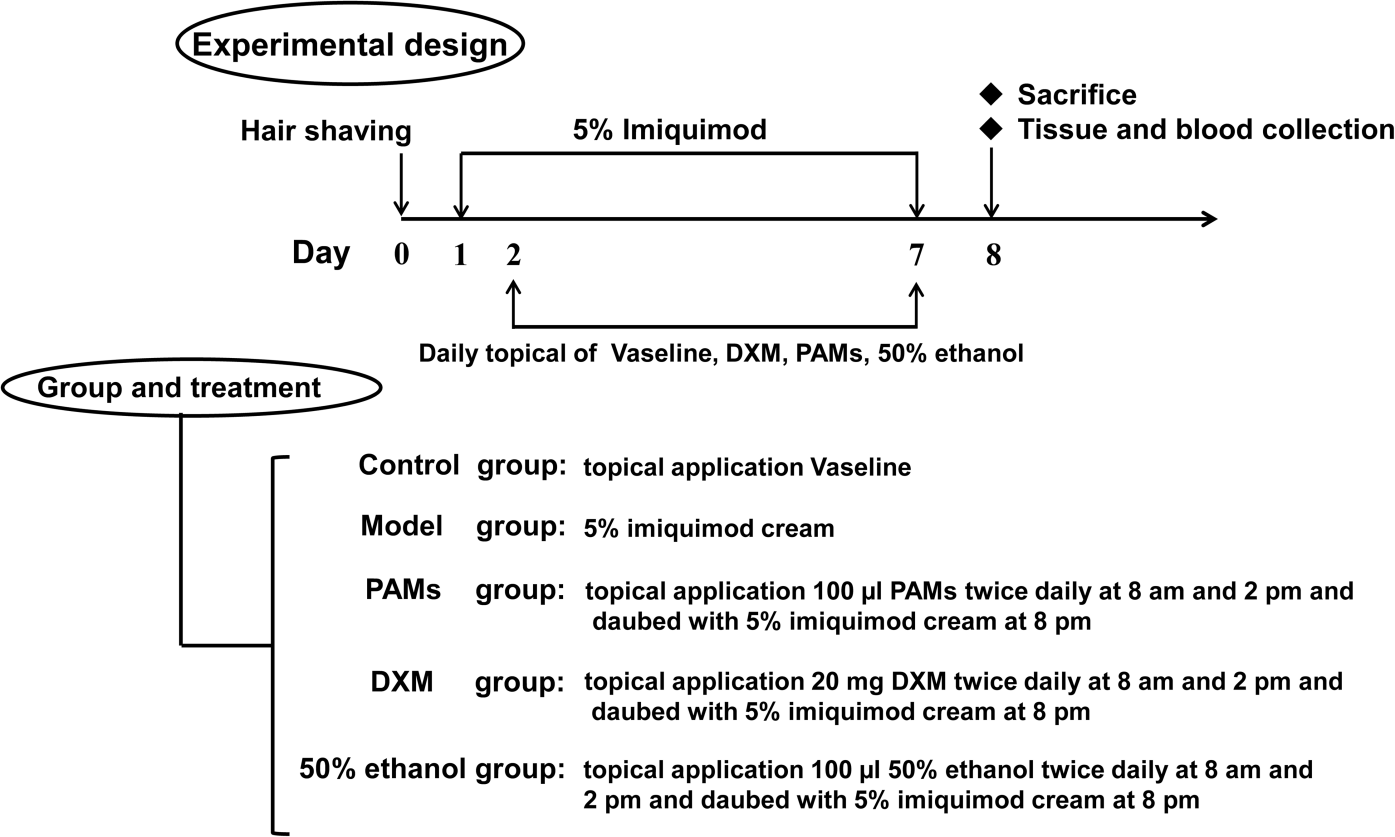
Diagram depicting the design of the imiquimod-induced skin-inflammation mouse model. BALB/c mice were divided into five groups (n = 6 per group): (1) Control group, (2) Model group, (3) PAMs group, (4) positive drug [Dexamethasone Acetate Cream (DXM)] group, and (5) 50% ethanol group.

The psoriasis-like skin inflammation mouse model was established by topical application of 62.5 mg of 5% imiquimod cream (Aldara; 3M Pharmaceuticals) to a 3.0 cm^2^ mouse shaved epidermal area for 7 consecutive days. In this experiment, the mice were randomly assigned into five groups, including the control group, model group, positive drug group [Dexamethasone Acetate Cream (DXM), Hubei Ketian Pharmaceutical Co., Ltd., Tianmen, China)], PAMs group, and 50.0% ethanol group. Each group consisted of six mice. In the control group, the shaved skins of the mice were treated with Vaseline alone twice a day at 8: 00 am and 2:00 pm. In the model group, the shaved skins were only treated with 5.0% imiquimod cream at 8:00 pm. In positive drug group, the shaved skins of the modeled mice were treated with approximately 20.0 mg DXM twice a day at 8:00 am and 2:00 pm and daubed with 5% imiquimod cream at 8:00 pm. In the PAMs group, the shaved skins were treated with approximately 100.0 μL of PAMs twice a day at 8:00 am and 2:00 pm and daubed with 5.0% imiquimod cream at 8:00 pm. In the 50.0% ethanol group, the shaved skins were treated with approximately 100 μL of 50.0% ethanol twice a day at 8:00 am and 2:00 pm and daubed with 5.0% imiquimod cream at 8:00 pm, 12 h after the last imiquimod treatment. Bloods and skin tissues were collected for further study. The diagram depicting the experimental design is shown in Fig 1.

To evaluate the severity of inflammation of the shaved back skin, the method of Psoriasis Area and Severity Index (PASI) described by van der Fits was introduced in our study [18]. Briefly, four important parameters of skin erythema, scaling, thickness and cumulative scores were scored. Each parameter was scored separately on a scale ranging from 0 to 4 according to the degree of the inflammation. The cumulative scores indicate the severity of inflammation.

### Histology and immunohistochemistry

The mouse shaved back skin samples were saved in 4.0% paraformaldehyde and embedded in paraffin. For histopathological examination, the sections (4.0 μm) were stained with hematoxylin and eosin (H&E) and observed under a light microscope (Olympus, Tokyo, Japan). Epidermal thickness was calculated by the average of six independent fields under the microscope. For immunohistochemistry examination, anti-mouse ICAM-1 antibody (10831-1-AP, Proteintech Group Inc, Wuhan, China) or anti-mouse Ki67 antibody (YM3064, Immunoway, Plano, Texas, USA) were used as the primary antibody at a 1:200 dilution. The sections were incubated with diluted biotin-conjugated goat anti-rabbit IgG at 4℃ for 50 min. Finally, they were developed using DAB, stained with haematoxylin and fixed using neutral balata. The sections were observed under a light microscope (Olympus, Tokyo, Japan).

### RNA isolation and quantitative real time PCR (RT-qPCR) in mice

Total mRNA was extracted from the back skin using TRIzol reagent after sacrificing the mice; Two microgram of total RNA was converted to cDNA with an iScript cDNA Synthesis kit. Quantitative real-time PCR was performed using SYBR Green Master Mix according to manufacturer's instructions. The sequences of the real-time PCR primers are shown in Table 3. Melting curve analysis was used to evaluate the primer specificity of the amplification. The gene *GAPDH* was used as the internal control. The relative mRNA expression of *IL-8*, *TNF-a*, *ICAM-1*, *IL-23* were calculated by normalizing to *GAPDH*.

**Table 3:** Primer sequences of mouse genes by quantitative real-time PCR.

Genes	Primer sequence	Tm
*ICAM-1*	F: 5’-AGACACAAGCAAGAAGACCACA-3’	60°C
	R: 5’-TGACCAGTAGAGAAACCCTCG-3’	
*TNF-a*	F: 5’-GCCCACGTCGTAGCAAACCAC-3’	60°C
	R: 5’-GCAGGGGCTCTTGACGGCAG-3’	
*IL-8*	F: 5’-GCTGTGACCCTCTCTGTGAAG-3’	60°C
	R: 5’-CAAACTCCATCTTGTTGTGTC-3’	
*IL-23*	F: 5’-TCCTCCAGCCAGAGGATCACCC-3’	60°C
	R: 5’-AGAGTTGCTGCTCCGTGGGC-3’	
*GAPDH*	F: 5’-GGGCTCTCTGCTCCTCCCTGT-3’	60°C
	R: 5’-CGGCCAAATCCGTTCACACCG-3’	

### Localization of NF-κB by immunofluorescence staining in mouse

The detection of NF-κB nuclear translocation was carried out following the instruction of the manufacturer. Briefly, the mouse back skin samples were first saved in 4.0% paraformaldehyde and embedded in paraffin, then 4.0 μm tissue sections were prepared, dewaxed and rehydrated. After fixing with 4.0% paraformaldehyde, permeabilized with 0.1% Triton X-100 for 10 min and blocked with 5% BSA for 1 h at room temperature, the sections were incubated overnight with anti-mouse p65 antibody (dilution 1:200, AN365, Beyotime Institute of Biotechnology, Nanjing, China) at 4℃. Later, they were washed twice with PBS and incubated with FITC-labeled secondary antibody (A0562, Beyotime Institute of Biotechnology, Nanjing, China) for 1.5 h at 37℃. Finally, each section was stained with 100.0 μl DAPI for 4 min, washed twice with PBS and observed under a fluorescence microscope. The translocation rate was counted from all fields.

### Statistical analysis

All experiments were performed in triplicates and were repeated at least three times. Data were analyzed using SPSS 19.0 software (IBM SPSS, Armonk, NY, USA) and R package. All graphical values were expressed as means ± SD. Student’s t-test was used to determine the statistical significance. A *p*˂0.05 value was considered significant and a *p*˂0.01 value was most significant.

## Results

### Quantitative analysis of the two main functional components in PAMs

**Fig 2:**
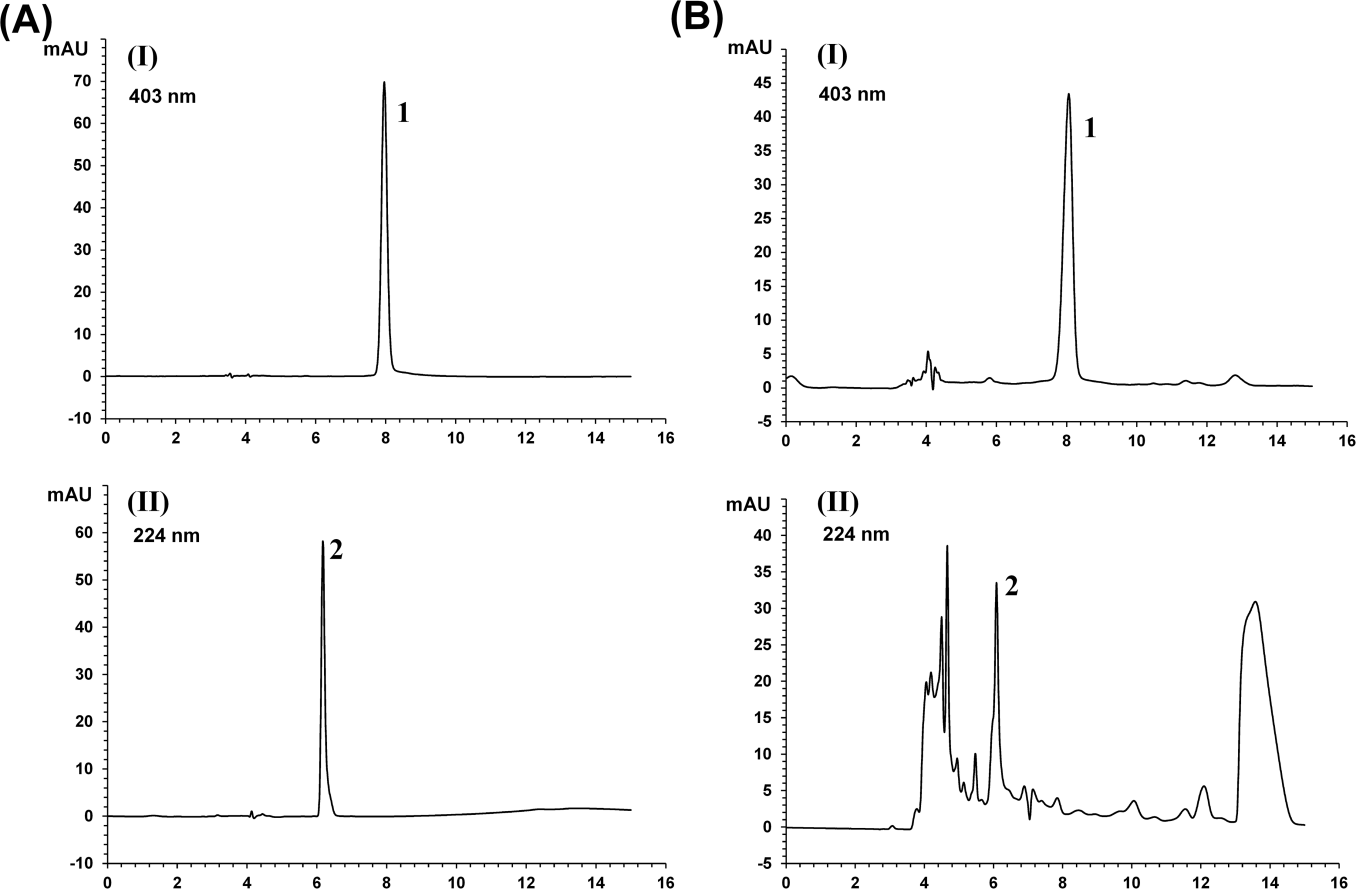
Representative HPLC chromatogram of two main active components of PAMs. a: Standard solution (A); b: PAMs sample (B). The detection wavelengths for hydroxysafflor Yellow A (1), and allantoin (2) in PAMs were at 403 and 224 nm respectively.

HPLC chromatograms were obtained using an isocratic flow under different conditions for the two main active components of hydroxysafflor Yellow A and allantoin in PAMs. Their contents were determined based on the peak areas. Using optimized chromatography conditions, each component was separated within 20 min. A representative HPLC chromatogram of PAMs is shown in Fig 2. The calibration curves of all compounds revealed good linearity with r^2^<0.99 for six different concentration ranges (S1 Table). The contents of hydroxysafflor Yellow A and allantoin in PAMs were 94.2±2.2, 262.9±12.5 μg/mL respectively.

### Effects of PAMs on TNF-α/IFN-γ-induced expression of cytokines/chemokines in HaCaT cells

CCK-8 assay was used first to determine the effects of dose-dependence curve of PAMs on the viability of HaCaT cells. Treatment with PAMs for 24 h at concentrations from 1.0% to 3.0% had no significant cytotoxic effects on HaCaT cells, however, PAMs over 4.0% concentration showed a significant cytotoxic effect on these cells (*p*<0.05) (see in S1 Fig). Therefore, we used PAMs at 3% concentration in subsequent cell experiments.

**Fig 3:**
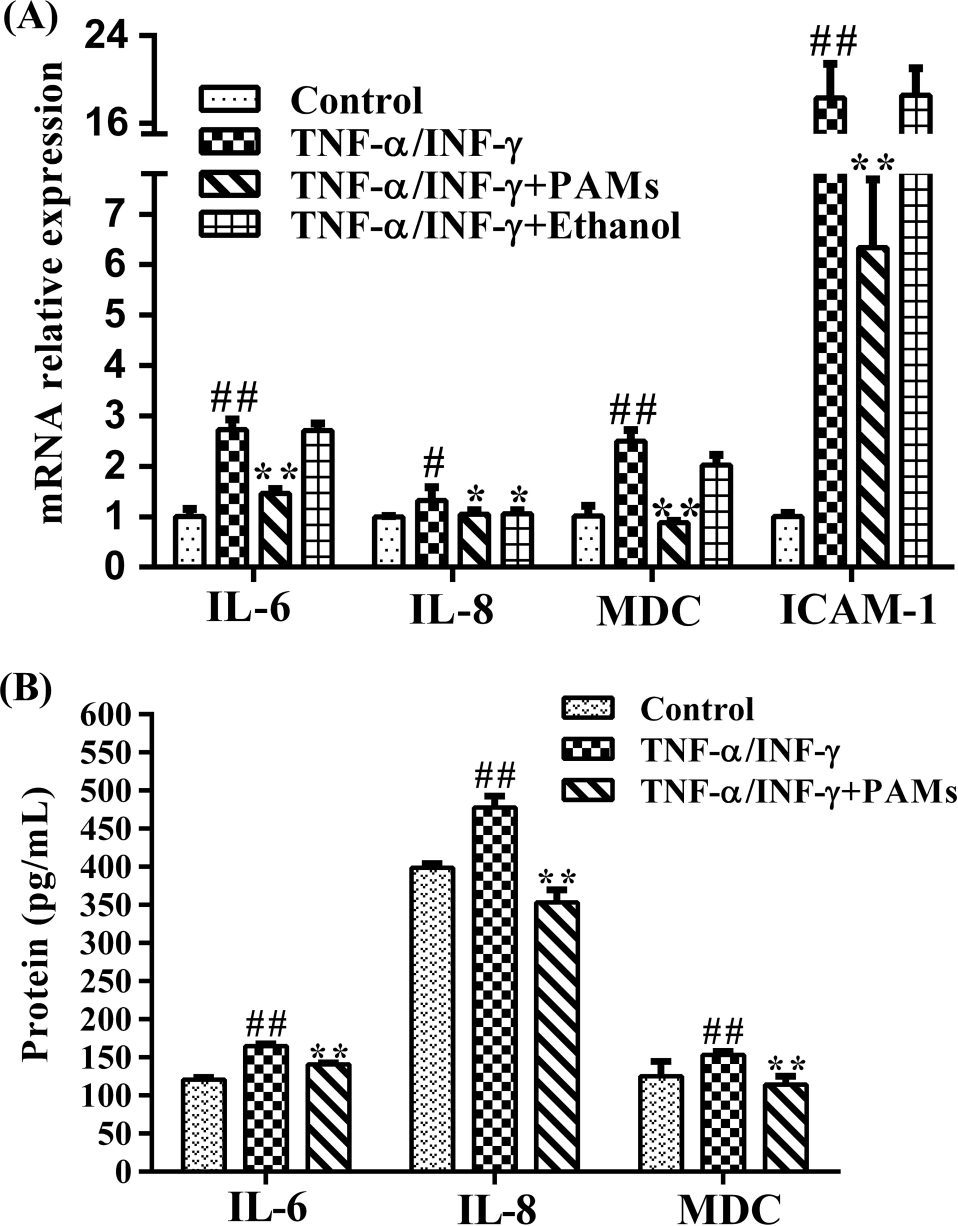
Effects of PAMs on the production of inflammatory cytokines in TNF-α and IFN-γ-induced HaCaT cells. A: Cells were treated with 3.0% PAMs, and TNF-α and IFN-γ (each 10 ng/mL) for 24 h. RT-qPCR was performed to determine the mRNA expression levels of *IL-8*, *IL-6*, *MDC* and *ICAM-1*. Values are expressed as mean± S.D of three independent experiments. B: Production of IL-8, IL-6 and MDC was measured in the culture supernatant of cells treated with 3.0% PAMs, and TNF-α and IFN-γ (each 10 ng/mL) for 24 h. ##*p* < 0.01 vs control cells; **p<0.01 *vs* TNF-α /IFN-γ-treated cells.

**Fig 4:**
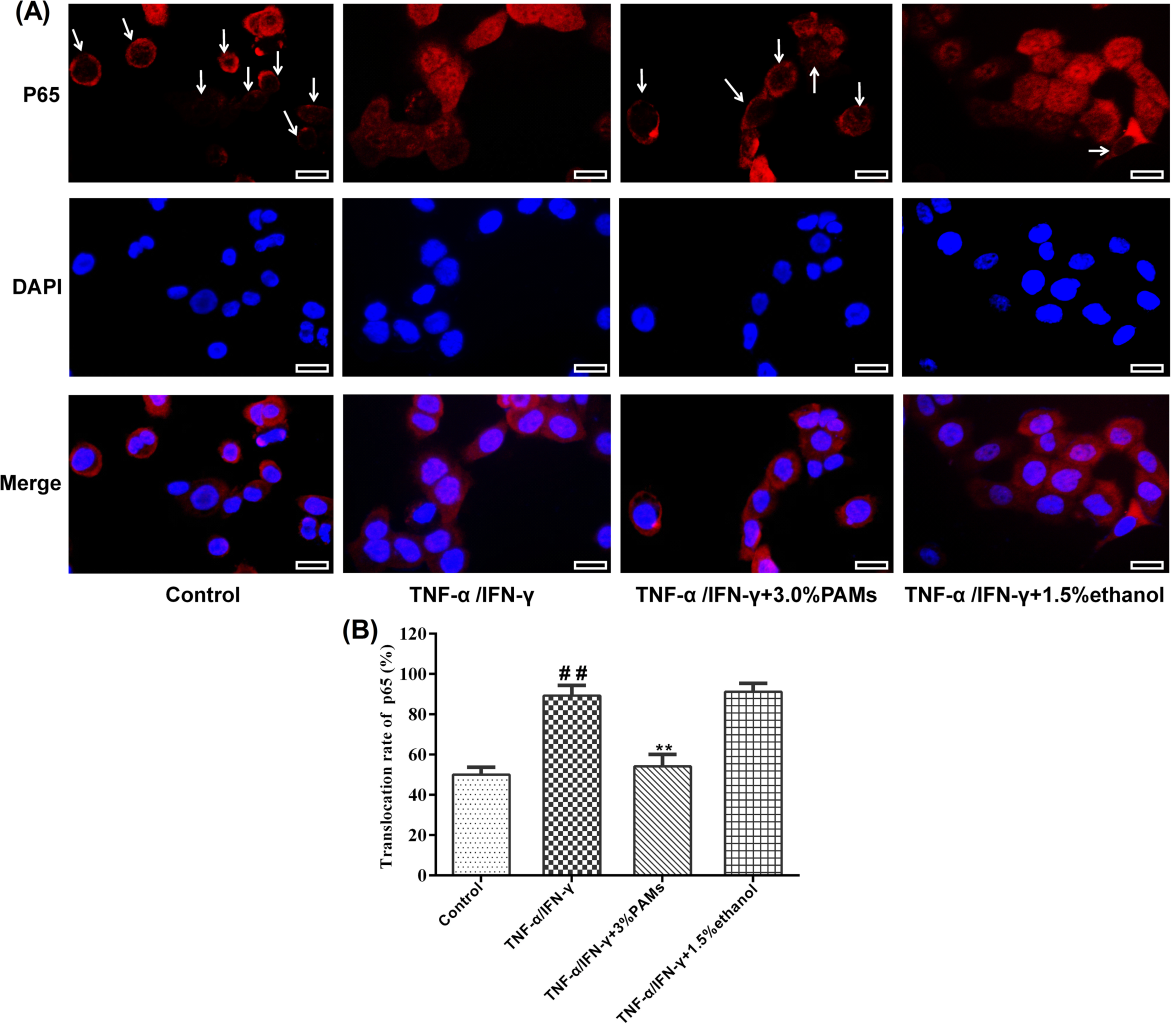
Inhibitory effects of PAMs on NF-κB activation and nuclear translocation in HaCaT cells. A: Cellular localization of p65 was analyzed by immunofluorescence staining. HaCaT cells were pretreated with or without 3.0% (v/v) PAMs or 1.5% (v/v) ethanol for 6 h and incubated with TNF-α and IFN-γ (each 10.0 ng/mL) for 30 min, later, the cells were incubated with anti-p65 and Cy3-conjugated secondary antibodies subsequently. Arrows indicate that NF-κB does not translocate to the nucleus. Images are representative of three independent experiments. B: The translocation rate was counted. ##*p* < 0.01 vs control group; **p < 0.01 vs model group. Bar = 50μm.

Inhibition of PAMs on the inflammatory cytokine production in TNF-α and INF-γ induced HaCaT cells was examined by both the cytokine mRNA expression in the cells and the cytokine production in the supernatants. As shown in Fig 3A, we found that 3.0% PAMs inhibited the relative mRNA expression of *MDC* (0.35-fold), *IL-6* (0.53-fold), *IL-8* (0.79-fold) and intercellular adhesion molecule-1(*ICAM-1*, 0.35-fold) as compared with those of TNF-α/IFN-γ treatment by RT-qPCR. The productions of cytokines in supernatants was consistent with the mRNA expression in cells, co-treatment of TNF-α and INF-γ significantly increased the contents of inflammatory cytokines of MDC, IL-6 and IL-8 while treatment with 3% PAMs significantly reduced their production in supernatants (Fig 3B). Although 1.5% ethanol showed a significant effect on the mRNA expression of *IL-8*, the effect on IL-8 was not significant. Moreover, as shown in Fig 4, in the nucleus, the red fluorescence of p65 protein was less intensive in TNF-α/IFN-γ-induced and PAMs-treated HaCaT cells than those of TNF-α/IFN-γ-induced HaCaT cells, suggesting that the activation of NF-κB was inhibited. As a negative control, 1.5% ethanol showed little effects on the inhibition of mRNA expression of *MDC*, *IL-6*, *ICAM-1*, and the activation of NF-κB.

### PAMs could ameliorate the imiquimod-induced psoriasis-like skin inflammation in mouse

**Fig 5:**
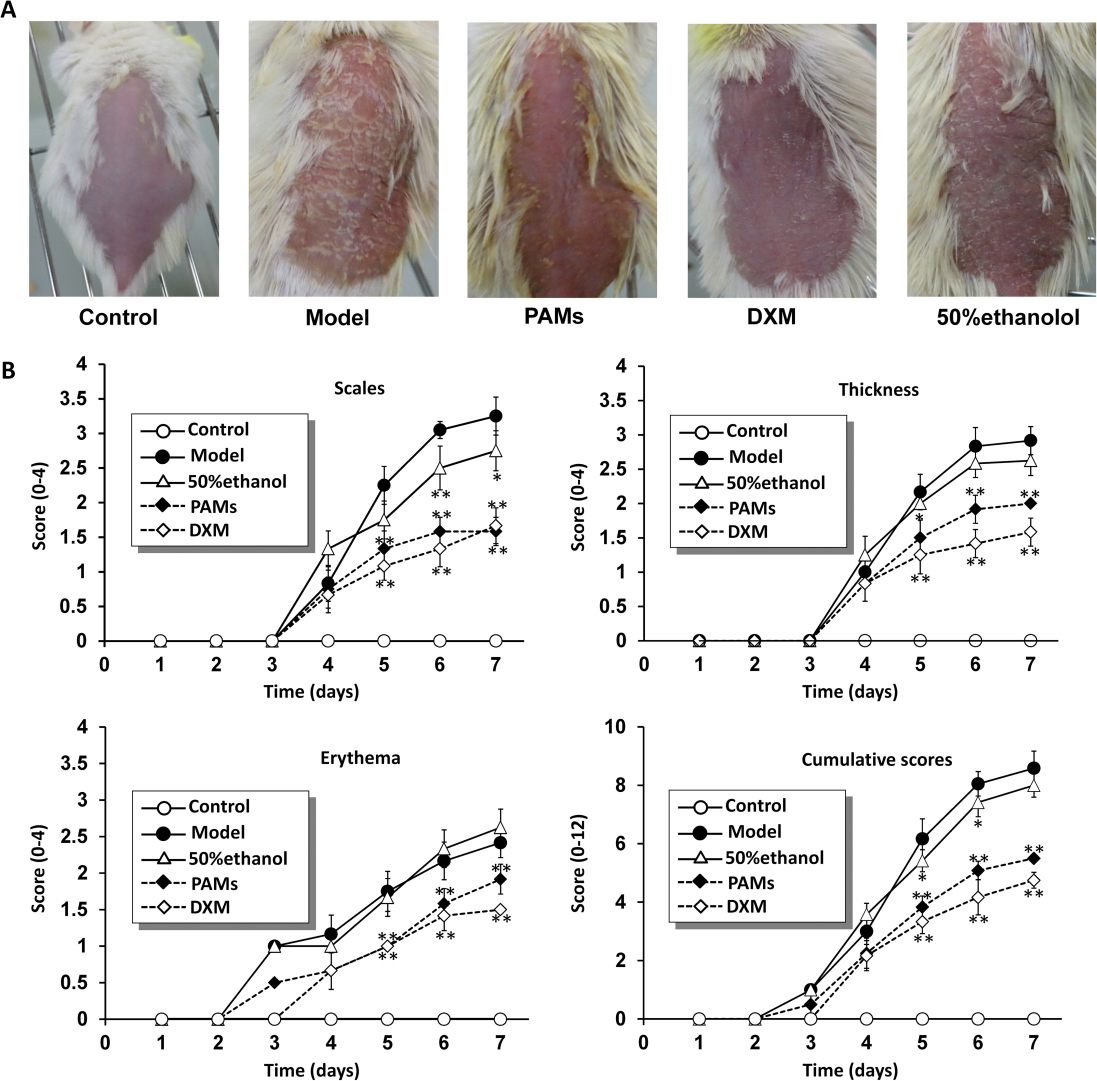
Effects of PAMs on erythema, scaling and thickening in imiquimod-induced mouse skin. A: Phenotypical presentation of mouse back skin after 6 days’ treatment. B: The scores of scales, thickness, erythema and the cumulative scores are shown for each group. The data are shown as mean± S.D (n = 6). Significant differences compared to model group: *p < 0.05.

After seven days’ treatment (see in S2 Fig), it was observed that PAMs could alleviate the psoriasis-like symptoms as compared with the control mice. Evaluation of inflammation severity using PASI is shown in Fig 5A and 5B, we could find that mice in the control group had smooth skin, as well as clear vessels under the back skin. In contrast, mice in the model group showed prominent erythema or flaky erythema in the back skin, almost all shaved skins were layered, obviously thickened and covered with apophysis. As expected, in the PAMs-treated group, skin lesions were obviously alleviated and there were statistically significant decreases in erythema, scaling, skin thickening as compared to those of the model group. The dexamethasone (DXM) group showed similar changes as compared to those of the PAMs group. However, skin lesions could not be significantly ameliorated in the 50% ethanol group.

**Fig 6:**
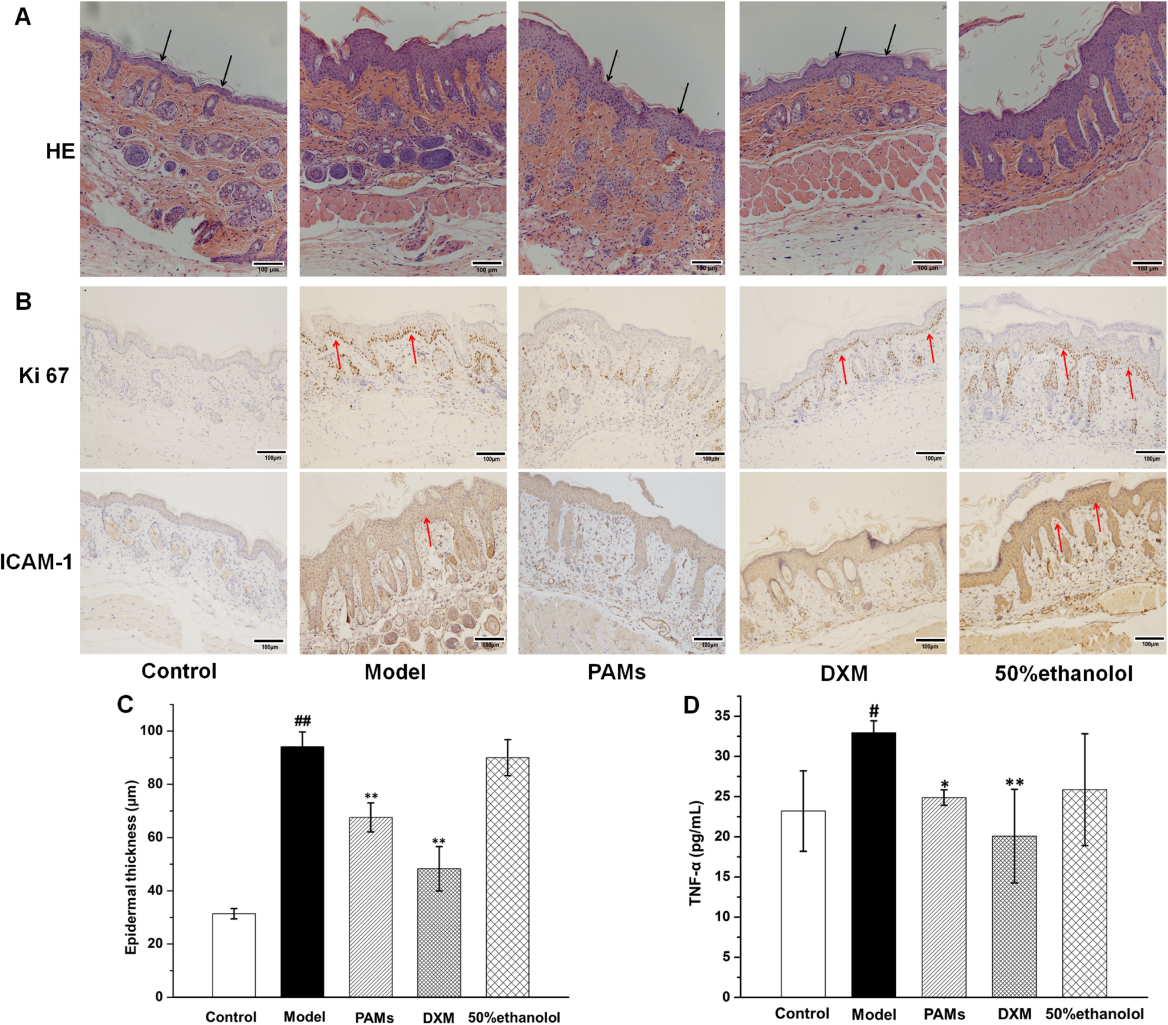
PAMs ameliorates the psoriasis-like symptoms and skin inflammation in imiquimod-induced mice. A: Histopathological investigation in each group (×100). Black arrows represent the presence of horny layer and granular layer. B: Immunohistochemistry analysis was performed for Ki67 and ICAM-1 protein of the back skin of psoriatic mice, PAMs treatment could effectively decrease the expression of Ki67 and ICAM-1 protein (×100). Red arrows represent the overexpression of Ki67 and ICAM-1 protein. C: Epidermal thickness was measured. Data are the mean values ± SD (n = 5). D: PAMs treatment led to the significant decrease of TNF-α levels in the serum of each group. #*p* < 0.05 *vs* control group; *p < 0.05 *vs* model group. Bar = 100 μm.

**Fig 7:**
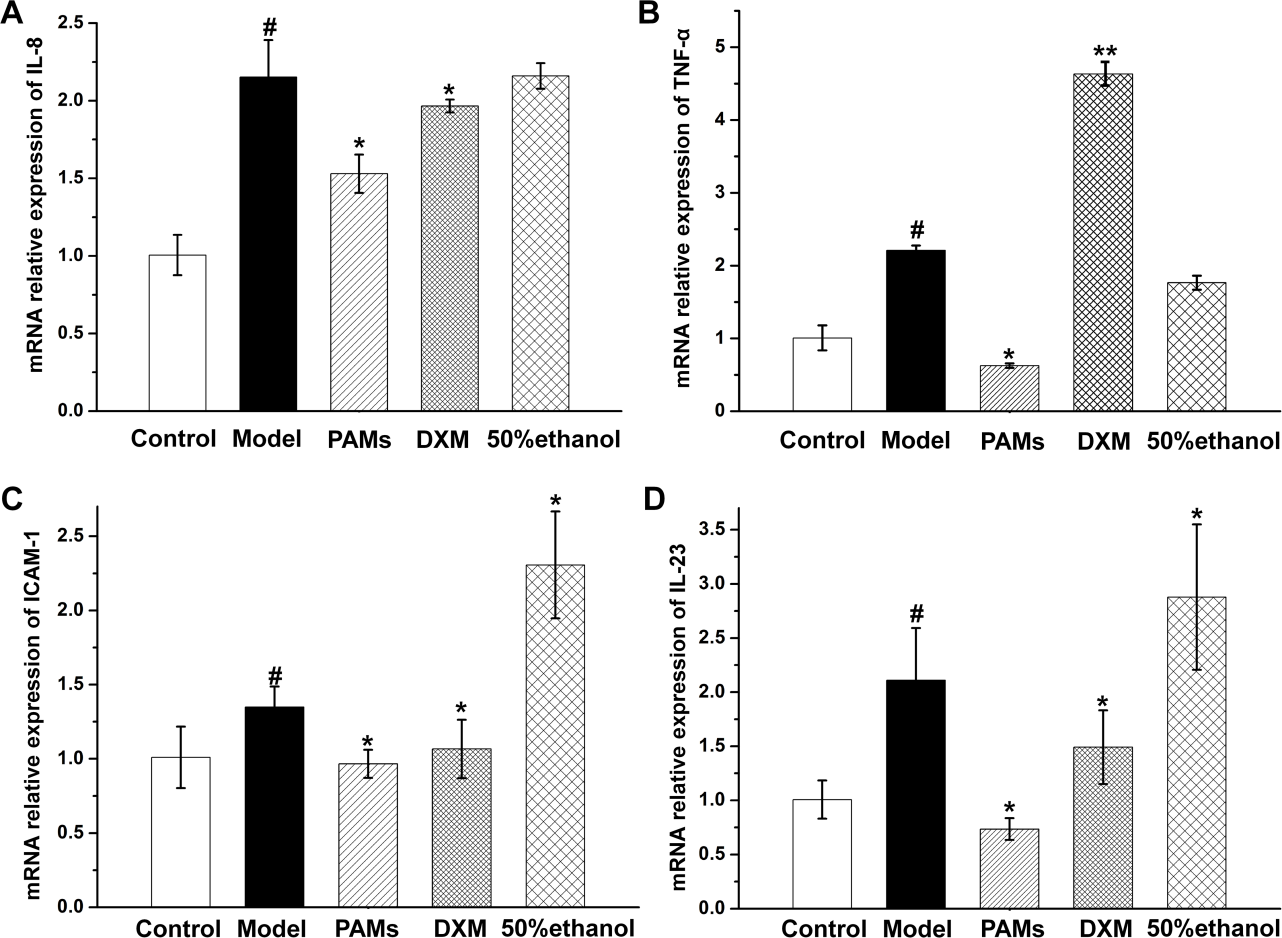
RT-qPCR of cytokines expression in mouse skin samples. RT-qPCR was performed to determine the mRNA expression levels of *IL-8*(A), *TNF-α*(B), *ICAM-1*(C) and *IL-23*(D). Values are expressed as mean ± S.D of three independent experiments. #*p* < 0.05 vs control group; *p < 0.05 vs model group.

**Fig 8:**
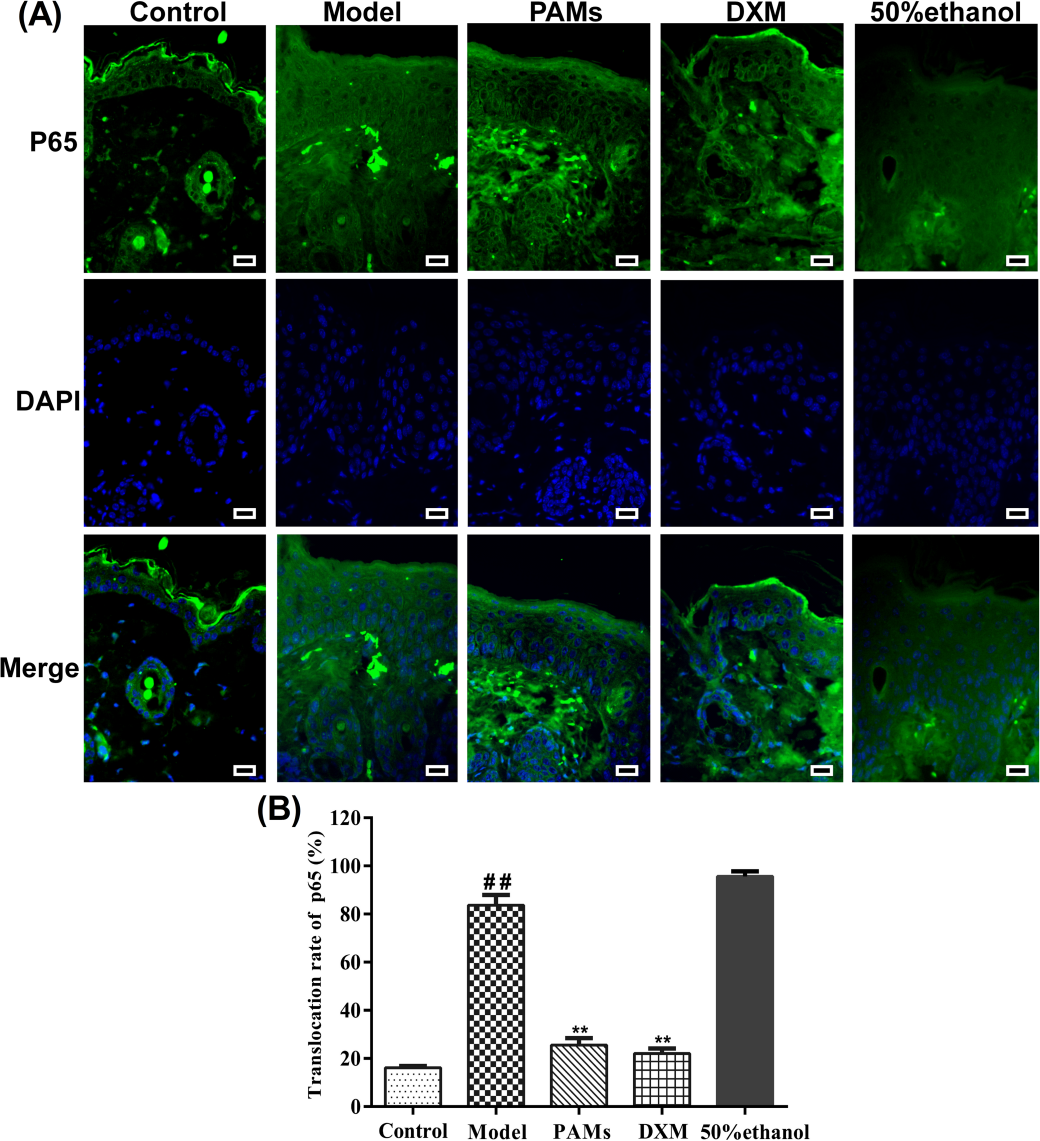
Effects of PAMs on NF-κB inactivation and nuclear translocation in imiquimod-induced mouse skin. A: Localization of NF-κB P65 protein was visualized in each group under a fluorescence microscope after staining with P65 antibody and FITC-labeled secondary antibody (green), the cell nuclei were stained with DAPI (×100). Images are representative of three independent experiments. B: The translocation rate was counted. #*p* < 0.05 *vs* control group; *p < 0.05 *vs* model group. Bar = 50 μm.

Based on histopathological analysis (Fig 6A), the epidermal layers of the skin lesions in imiquimod-induced model mice were found to be thicker than those of the normal control, the increased number of keratinocytes in basal cell layer caused acanthosis in the skin. In addition, the horny layer and granular layer were absent in the imiquimod-induced mice, all of them showed the same histological characteristics as psoriasis-like skin. PAMs and DXM could weaken most of these changes; in contrast, 50% ethanol showed little effect on these characteristics. The results suggested that PAMs could effectively delay the onset of imiquimod-induced inflammation in mice and ameliorate the skin lesions of imiquimod-induced psoriasis. Further studies revealed that the effect was tightly related to the reduction of Ki67-positive cells and the expression of ICAM-1 protein was significantly inhibited in the PAMs treated group (Fig 6B). Quantitative analysis showed that imiquimod-induced epidermal thickening (94.1±5.5μm) was most significantly inhibited by PAMs (67.6±5.5μm, *p*˂0.01, Fig 6C). Moreover, PAMs significantly decreased the TNF-α level to 24.88±0.96 pg/mL as compared to 32.95±1.48 pg/mL in imiquimod-induced mouse model in blood (Fig 6D). Furthermore, PAMs significantly inhibited the gene expression of *TNF-α* (0.28-fold), *IL-23* (0.35-fold), *IL-8* (0.71-fold) and *ICAM-1* (0.72-fold) as compared with imiquimod-induced mouse model in skin lesions by RT-qPCR (Fig 7). Finally, as shown in Fig 8, the green fluorescence of P65 was intensive in the cell nucleus of the skin sections of model group, while the intensity was inhibited when topical application of PAMs, suggesting that the activation of NF-κB was inhibited by PAMs.

## Discussion

It is now well accepted that herbal medicines may provide new therapeutic approaches and play key roles in treatment of inflammatory skin diseases [19]. In this study, we report a Chinese folk medicinal preparation (PAMs) on its anti-inflammatory effects both *in vitro* and *in vivo*. As TCM, it has been shown that *L*.*erythrorhizon* and *S*. *indicum L* had strong anti-inflammatory activity [20,21]. There were also reports about the active components isolated from such plants as *L*.*erythrorhizon*, *C*. *tinctorius*, having significant inhibitory effects on skin diseases [21,22]. However, this is the first report of this ancient and complex folk formula showing such remarkable and therapeutic effects on inflammatory psoriasis-like skin diseases. In addition, there were successful cases of its application in the treatment of psoriasis patients. Here, we firstly quantified the two main compounds of hydroxysafflor Yellow A and allantoin, the active components of *C*. *tinctorius*, *L*. *erythrorhizon* respectively by HPLC­PDA. The results disclosed that the functional components of hydroxysafflor Yellow A and allantoin were abundant in PAMs. Therefore, at least the purity and concentration of these two compounds should serve as important indices for the quality control of PAMs.

Keratinocytes are the main epidermic cells and play a key role in immune response by expressing Toll-like receptor (TLR) molecules [23]. There is an abundance of evidence to suggest that proinflammatory cytokines (IL-6) and chemokines (IL-8, MDC) produced by keratinocytes may be responsible for skin inflammatory diseases including psoriasis [7,8]. Due to the similar characteristics to primary keratinocytes [24], HaCaT cells were often used in pharmacological study for potential skin drug development [9]. In this work, we investigated the effects of PAMs on TNF­α/IFN­γ-induced inflammation in keratinocytes, the well-known model for the production of inflammatory cytokines and adhesion molecules in skin diseases [25]. PAMs was found to significantly inhibit the mRNA expressions of *MDC*, *IL-8* and *IL-6*, these results were further supported by the reduced production of them in supernatants except for IL-8. PAMs also suppressed the expression of *ICAM-1*as the expression of *ICAM-1* was significantly increased in HaCaT cells when stimulated with TNF-α/IFN-γ [26,27]. Moreover, we demonstrated that PAMs could ameliorate imiquimod-induced psoriasis-like skin inflammation in the mouse model. Imiquimod, a ligand for TLR7/8, has been widely used for the topical treatment of anal warts, condyloma acuminata, basal cell carcinoma and solar keratosis [28]; however, it can also exacerbate psoriasis in well-controlled patients [29]. Consistently, research findings indicated that topical application of imiquimod on the mice can induce psoriasis-like skin inflammations [30]. Therefore, this animal model can be used to analyze the pathogenesis of psoriasis by showing similar characteristics to human psoriasis such as erythema, scaling and hyperkeratosis in epidermis [31]. In our mouse experiment, in order to eliminate the influence of ethanol in PAMs, 50% ethanol was also employed as a control just as was done in previous cell experiment. As expected, 50% ethanol could not, but PAMs could significantly decrease the skin thickness, inflammatory reactions and repair skin lesions in imiquimod-induced mice. In addition, as we know, hyperplastic basal, suprabasal keratinocytes are the main reasons for skin thickness, a proliferation marker of Ki67 represents an overexpression in psoriatic skin lesions [10], Ki67 was introduced and the presence of Ki67 positive cells was consistent with the PASI scores in all our experimental groups.

In this study, we not only detected IL-8 and ICAM-1 which we assayed in the cell analysis, but also determined other important cytokines such as TNF-α and IL-23. ICAM-1 can be strongly produced and plays a key role in pathogenesis of psoriasis [32]. IL-8 and TNF-α are also involved in the course of psoriasis [33,34]; high levels of TNF-α associated with NF-κB activation has been found in psoriatic patients [10]. IL-23, which is overproduced by keratinocytes and dendritic cells in psoriatic lesions [33] is closely associated with acanthosis in psoriasis [35]. As compared to those of the control group, the results of RT-qPCR also indicated a significant increase in the mRNA levels of *IL-8*, *IL-23*, *TNF-α* and *ICAM-1* in the skin lesions of imiquimod-induced psoriasis-like mice from the model groups, however, their mRNA levels are significantly decreased in the PAMs treated mice. Dexamethasone, which has been commonly used for atopic treatment of psoriasis and dermatitis [36] and here was used as positive control, it also showed significantly lowering effects for the mRNA levels of *IL-8*, *IL-23*, and *ICAM-1*, except for *TNF-α*. We should emphasize that although the overexpression of *TNF-α* was found only in tissue samples, its level in serum is actually lower compared to those of model groups. As a negative control, ethanol treatment increased the expression of *ICAM-1* and *IL-23* as compared to those of model group. It indicates ethanol may enhance the production of ICAM-1 and IL-23 in this negative group. Taken together, these results corroborated well with our overall conclusions.

**Fig 9:**
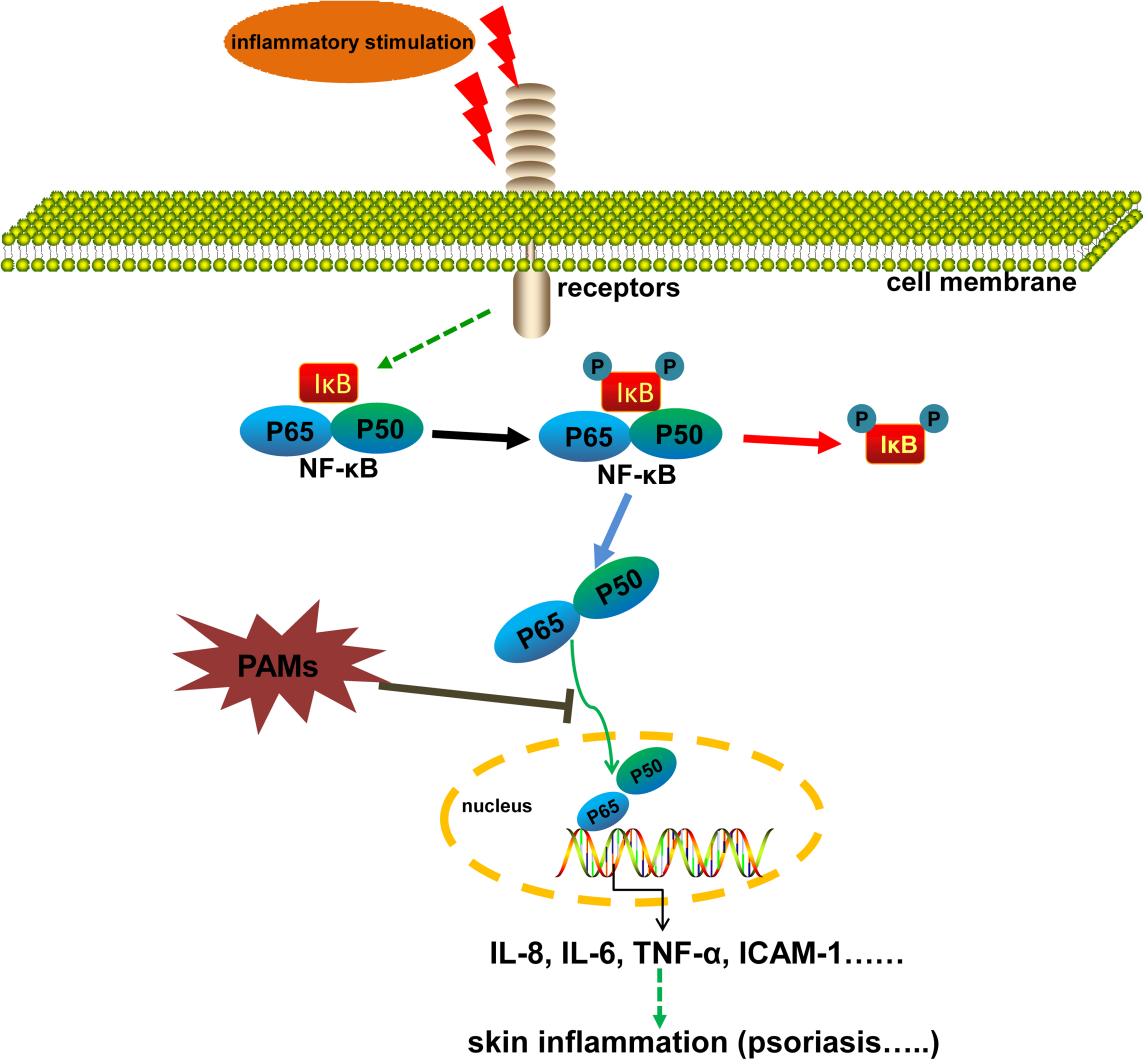
The proposed mechanisms of the inhibition of imiquimod-induced psoriatic inflammation in mouse by PAMs.

Finally, we studied if the inhibitory effects of PAMs on inflammation are correlated to the inhibition of NF-κB signaling pathway in cells. NF-κB is an inducible transcription factor and the inactive NF-κB is mainly located in the cytoplasm. Upon stimulation, NF-κB is activated by the translocation of p65 into the nucleus and the phosphorylation of IκB. NF-κB is thought to play a key role in inflammatory signaling pathways by regulation of many cytokines [9,37]. In the current study, we found that it could regulate the production of TNF-α, IL-8, IL-23 and ICAM-1 associated with psoriasis. Based on the localization of the p65 subunit, the activity of NF-κB could be clearly detected. Here we found that PAMs treatment inhibited the NF-κB signaling by significantly preventing the p65 translocation into the nucleus. The results of the immunofluorescence staining of mouse tissues were also consistent with histological and immunohistochemical observations on acanthosis or keratinocyte proliferation. Taking together, we propose the possible mechanisms for the inhibition of imiquimod-induced psoriasis-like skin disease in mice by PAMs as shown in Fig 9. PAMs blocks the activation of NF-κB induced by inflammatory stimuli, which results in the amelioration of inflammatory skin lesions.

## Conclusions

We reported a Chinese ancient formula and folk medicine preparation, named “natural plant antimicrobial solution” (PAMs) on its curative effect for skin inflammatory diseases. In addition to its known effects on microbial inhibition, pain-releasing and wounds healing, here we further qualified the products and found PAMs also had strong inhibitory effect for inflammatory skin lesions. We demonstrated the anti-inflammatory functions and proposed the mechanisms for the amelioration of psoriasis-like symptoms by PAMs both in TNF-α/IFN-γ-induced HaCaT cells and imiquimod-induced psoriasis-like mouse. Further studies are in progress to search other bioactive components which could enhance the anti-inflammatory mechanisms of PAMs on skin inflammatory diseases including psoriasis.

## Supporting information

S1 Fig Cytotoxicity of PAMs in HaCaT cells. (TIF)

S2 Fig Phenotypical presentation of shaved mouse back skin from 2–7 days of treatment in each group. (TIF)

S1 Table Regression equations, linearity and correlation coefficient for two compounds of PAMs. (DOCX)
