# Correction: PAMs ameliorates the imiquimod-induced psoriasis-like skin disease in mice by inhibition of translocation of NF-κB and production of inflammatory cytokines

**DOI:** 10.1371/journal.pone.0198501

**Published:** 2018-05-30

**Authors:** Rongkun Dou, Zongying Liu, Xue Yuan, Danzhou Xiangfei, Ruixue Bai, Zhenfei Bi, Piao Yang, Yalan Yang, Yinsong Dong, Wei Su, Diqiang Li, Canquan Mao

In Table 3, the primer PCR sequence of IL-8 is incorrectly shown for IL-18 (NM_008360.2). Please see the primer PCR sequence of IL-8 (NM_011339) in the corrected Table 3 here.

**Table 3:** Primer sequences of mouse genes by quantitative real-time PCR.

Genes	Primer sequence	Tm
*ICAM-1*	F: 5’-AGACACAAGCAAGAAGACCACA-3’	60°C
	R: 5’-TGACCAGTAGAGAAACCCTCG-3’	
*TNF-a*	F: 5’-GCCCACGTCGTAGCAAACCAC-3’	60°C
	R: 5’-GCAGGGGCTCTTGACGGCAG-3’	
*IL-8*	F: 5’- CGGCAATGAAGCTTCTGTAT -3’	60°C
	R: 5’- CCTTGAAACTCTTTGCCTCA -3’	
*IL-23*	F: 5’-TCCTCCAGCCAGAGGATCACCC-3’	60°C
	R: 5’-AGAGTTGCTGCTCCGTGGGC-3’	
*GAPDH*	F: 5’-GGGCTCTCTGCTCCTCCCTGT-3’	60°C
	R: 5’-CGGCCAAATCCGTTCACACCG-3’	
