# Supplementary figures and images for: PAMs ameliorates the imiquimod-induced psoriasis-like skin disease in mice by inhibition of translocation of NF-κB and production of inflammatory cytokines
Source: PLoS One. 2017 May 2;12(5):e0176823. doi: 10.1371/journal.pone.0176823 (PMC5413058; doi:10.1371/journal.pone.0176823)

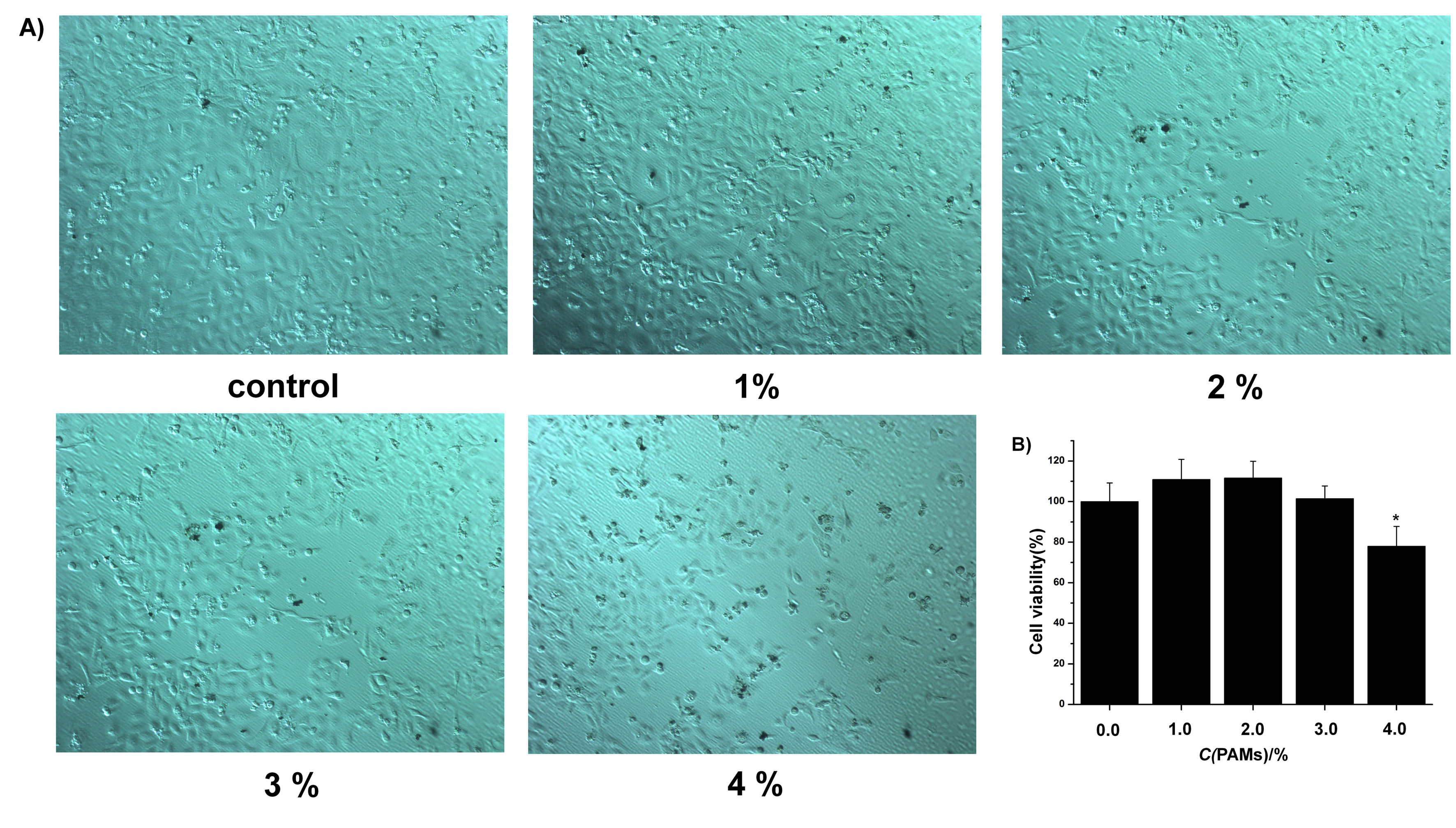

Supplement: S1 Fig — (TIF) [file pone.0176823.s001.tif]

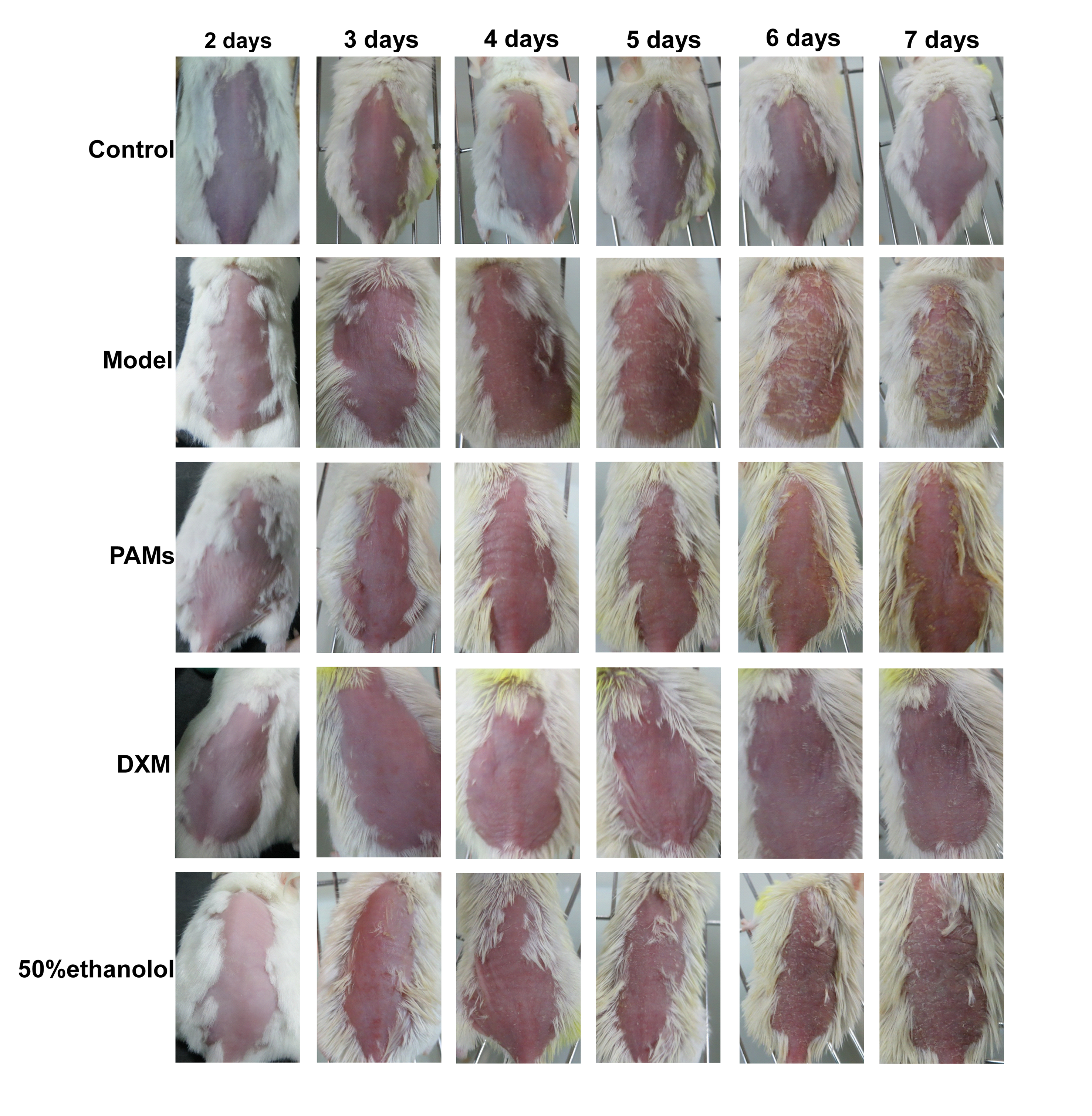

Supplement: S2 Fig — (TIF) [file pone.0176823.s002.tif]
