# Supplementary material for: PAMs ameliorates the imiquimod-induced psoriasis-like skin disease in mice by inhibition of translocation of NF-κB and production of inflammatory cytokines
Source: PLoS One. 2017 May 2;12(5):e0176823. doi: 10.1371/journal.pone.0176823 (PMC5413058; doi:10.1371/journal.pone.0176823)
Supplement: S1 Table — (DOCX) [file pone.0176823.s003.docx]

**S1 Table. Regression Equations, Linearity and Correlation Coefficient for Two Compounds of PAMs.**

| Compound | Linear range (μg/ml) | Regression equation^a)^ | Correlation coefficient  (*r*^2^) |
| --- | --- | --- | --- |
| Hydroxysafflor yellow A | 20 ~100 | y = 8.4162x + 34.116 | 0.9965 |
| Allantoin | 60~160 | y = 3.5109x + 15.741 | 0.9978 |

1. *y*: peak area (mAU) of compounds; *x*: concentration (*μ*g/mL) of compounds

**.**
